# Assessment of X Chromosome Centromere Instability in Alzheimer’s Disease: A Quantitative FISH Approach

**DOI:** 10.3390/cimb47060420

**Published:** 2025-06-05

**Authors:** Biljana Spremo-Potparević, Petar Popović, Dijana Topalović, Andrea Pirković, George Perry, Lada Živković

**Affiliations:** 1 Department of Pathobiology, Faculty of Pharmacy, University of Belgrade, 11000 Belgrade, Serbia; petar.popovic@pharmacy.bg.ac.rs (P.P.); dijana.topalovic@pharmacy.bg.ac.rs (D.T.); lada.zivkovic@pharmacy.bg.ac.rs (L.Ž.); 2 Department of Biology of Reproduction, Institute for the Application of Nuclear Energy, 11000 Belgrade, Serbia; andrea.pirkovic@inep.co.rs; 3 College of Sciences, The University of Texas, UTSA, San Antonio, TX 78249, USA; george.perry@utsa.edu

**Keywords:** Alzheimer’s disease, premature centromere division, X chromosome, chromosomal instability, fluorescence in situ hybridization

## Abstract

Chromosomal instability in Alzheimer’s disease (AD) neurons has been previously reported. This pilot study aimed to establish a quantitative technique for assessing X chromosome centromere signals using fluorescence in situ hybridization (FISH). Hippocampal brain tissue was collected at autopsy from sporadic AD patients and age- and gender-matched controls. FISH was utilized to detect and measure the intensity of hybridization signals for X chromosome centromeres in the interphase nuclei of hippocampal brain cells. The premature centromere division (PCD) phenomenon, marked by a close bipartite signal appearing as two separated FISH spots, was examined to see if the hybridized DNA amount in each spot matched the expected centromere DNA amount. The technique effectively distinguished between PCD+ and PCD− signals. The average PCD frequency of the X chromosome in the AD group was 7 ± 1%, compared with 3.2 ± 0.84% in the controls. This quantitative approach supports qualitative analyses of FISH centromere spots, reinforcing findings of chromosomal instability in AD. The presence of a double signal at the centromere of a single X chromosome indicates re-entered cell cycles, DNA replication, and PCD in hippocampal neurons. This technique provides a reliable method for identifying PCD + signals and contributes to understanding chromosomal instability in AD.

## 1. Introduction

Alzheimer’s disease (AD) is a complex and progressive age-related neurodegenerative disorder. Most age-related neurodegenerative diseases present different brain pathologies and clinical features, but they all share a common trait: a reduced number of neurons in specific parts of the brain [1]. The cytopathology of AD is very complex, and the identification of AD biomarkers is a highly relevant research area with many different approaches being explored [2]. Constant research of new biomarkers is of great importance for helping with the early diagnosis of pathological changes and improving the ability to monitor the course of AD disease and treat it [3]. To date, no reliable biomarker has been established that enables the diagnosis and/or assessment of the degree of risk of AD in susceptible individuals. Understanding the mechanisms behind neuronal loss and the cytopathological processes that precede it remains a major challenge for researchers.

One potential cell mechanism is the generation of neuronal aneuploidy as a consequence of neuron cell-cycle reentry reported in Alzheimer’s brains [4,5,6,7]. Between the end of DNA replication in the S phase of the cell cycle and the time when the segregation of replicated genetic material begins in the mitotic anaphase, two sister chromatids of the chromosome are tightly held together in the centromere region. Metaphase to anaphase transition is a point of no return, characterized by the segregation of chromosomes as a non-random process, hierarchically ordered and genetically controlled. Evidence suggests that the chromosome malsegregation presented in AD patients is related to neurodegeneration [4,5,6,7]. A significant increase in chromosome instability (CIN), as well as the presence of aneuploidy and a phenomenon of premature centromere division (PCD), has been observed in AD brain neurons and peripheral tissues [8,9,10,11,12].

PCD represents a loss of control over the sequential separation and segregation of chromosome centromeres that happens in the interphase of the cell cycle in the S period after DNA replication [13]. Our previous results confirmed that brain cells in the frontal cerebral cortex re-enter the cell cycle and that PCD during the interphase after replication leads to genome instability.

Chromosomal instability, especially alterations of the cell cycle and chromosome segregation patterns, may be a key feature leading to the apoptotic degradation of neurons in AD [14,15,16,17]. Furthermore, research indicates that brains affected by AD exhibit a two-fold increase in X chromosome aneuploidy in the hippocampus and cerebrum, suggesting that altered sex chromosome dosage contributes to genomic variations in neuronal cells. This finding is supported by evidence showing distinct differences between sex chromosomes and autosomes in the dorsolateral prefrontal cortex, with X chromosome aneuploidy linked to a faster rate of cognitive decline—a key feature of AD—which may play a role in both aging and disease progression [17,18]. The role of sex chromosomes in AD might be especially interesting, bearing in mind that two-thirds of clinically diagnosed cases of dementia and AD are women. Several studies have reported that X chromosome alterations indicate malsegregation during cell cycle events in AD female patients [11,12,13,14]. It has been shown that the PCD of the X chromosome is substantially present in the interphase nuclei of the frontal cerebral cortex neurons of AD female brains, as detected by the fluorescence in situ hybridization method (FISH) and manifested as close-paired fluorescent dots [13].

Given the frequent occurrence of X chromosome alterations in AD, it is beneficial to implement new approaches for assessing X chromosome imbalances (and other chromosomes) in interphase nuclei. FISH targeting the centromere region of the X chromosome is the method of choice for verifying centromere instability in interphase nuclei.

FISH is extensively utilized in prenatal diagnosis, cancer diagnostics, and the detection of copy number variants (CNVs). Despite its widespread use and enhancements since the early 1980s, FISH still faces unresolved issues, particularly in interpreting results during chromosome identification when using interphase FISH for various diagnostic and research applications. FISH artifacts can greatly diminish the results’ resolution due to factors like interphase chromosome over-positioning, low hybridization efficiency, etc. [19]. Methods for quantitative analysis and the differentiation of individual FISH signals from artifacts are essential for addressing these issues. Despite ongoing interest, standardized protocols for FISH signal quantitative assessment remain absent, and challenges include reproducibility, signal irregularity, and background autofluorescence. The significant development of the method was achieved by Iourov et al. [20], who demonstrated distinguishing between active and inactive X chromosomes by quantitatively assessing FISH signals. The authors used a chromosome X-specific alphoid DNA probe for studying X chromosome inactivation and showed that this approach can enhance the technique’s efficiency by up to 47.5% due to the detection of chromosomal heteromorphism with minimal differences in alphoid DNA content between homologous chromosomes [20]. Furthermore, these initial molecular cytogenetic assays for X inactivation fully confirmed the results obtained from subsequent analyses using a restriction/quantitative PCR-based assay.

Based on this approach as a highly efficient tool for use in studies of chromosomes alterations in interphase nuclei, we performed FISH targeting for the centromere region of the X chromosome to the interphase nuclei of hippocampus neurons from sporadic cases of AD women postmortem.

For the quantitative analyses of FISH signals, an image analysis system was used to determine whether duplicated centromere spot signals represent the PCD phenomenon. To the best of our knowledge, this is the first time quantitative analysis has been performed on postmortem brain samples to determine centromere instability in the neurons of AD patients.

Given the established correlation between PCD occurrence in neuronal cells and peripheral blood lymphocytes of Alzheimer’s patients, we suggested that X chromosome PCD might serve as a potential biomarker for the early diagnosis of Alzheimer’s disease.

## 2. Materials and Methods

### 2.1. Subjects and Sampling

Hippocampal brain tissue was collected at autopsy from five female AD patients (ages 74–79 years) with clinical parameters consistent with a diagnosis of sporadic AD and five age-matched female controls (ages 73–78 years) with no evidence of neurodegenerative disease, according to approved protocols and with written family consent. Tissue samples were preserved in formalin-fixed, paraffin-embedded (FFPE) blocks and stored at a cool, dry, dark place with consist temperatures between 15 °C and 25 °C in storage boxes, and all samples were collected within a year for the purpose of this study. Permission from the Ethical Committee of the Clinical Centre of Serbia, protocol No. 2494/7, was obtained (permission granted on 13 November 2013).

### 2.2. FISH Analysis of Premature Centromere Division of the X Chromosome in Interphase Nuclei

Human-anonymized postmortem brain tissue from the hippocampus of sporadic AD patients and age-matched controls was routinely formalin-fixed, paraffin-embedded, and sectioned at 4 μm. In the hematoxylin and eosin (H&E)-stained sections, the neuronal cells were distinguished, and verification was performed by pathologists at the University Clinical Center of Serbia (Department of Pathology, histopathology, and medical cytology), and the fields of interest were photographed (archived specimens available at the clinical repository for non-research purposes). A serial of cross sections were prepared from the same section of brain tissue with the purpose of confirming that FISH analyses with a centromere probe of the X chromosome were done on the same cells and the sections were co-localized with the H&E-stained sections. The same approach has been described in our previous research on cerebral cortex neurons [21]. Histological preparation of hippocampus sections embedded in paraffin was performed by immersing the sections into xylene (Sigma–Aldrich, Munich, Germany) at room temperature. Sections were rehydrated in graded ethanols (100% to 70%), then washed in phosphate saline buffer (PBS) pH 7.4 (Sigma–Aldrich, Munich, Germany). The slides were immersed into H_4_BNa 10 mg/mL PBS, followed by washing in PBS, deionized water (deH_2_O), and 0.1 N hydrochloric acid. After neutralization with deH_2_O, preparations were immersed into a 2xSSC buffer (Sigma–Aldrich, Munich, Germany) and 8% (*v*/*v*) NaSCN at 80 °C. The slides were then rinsed with deH_2_O and 2xSSC buffer before being immersed into a trypsin solution (1 mg/mL saline solution) at 37 °C. Trypsin activity was blocked by immersion into 0.5% newborn calf serum solution (Thermo Fisher Scientific, Waltham, MA, USA). The slides were dehydrated in graded ethanols (70% to 100%) and air-dried. Cytocell (Cambridge, UK) directly labeled (FITC) α-satellite pancentromeric probes for the X chromosome were applied to the preparations. The manufacturer’s co-denaturation and hybridization instructions were followed. After hybridization, the slides were washed with 2xSSC 0.05% Tween (Sigma–Aldrich, Munich, Germany), dehydrated in a series of ethanol percentages, and counterstained with DAPI-Antifade (Life Technologies, Eugene, OR, USA).

In the interphase nuclei of neurons from the hippocampus of the AD female patients and the female controls, one dot-like signal was verified in one of the X chromosomes (PCD−) and two separate, close dot-like signals (PCD+) in the centromere region of the other X chromosome. To obtain PCD+ signals, the cell must first transit from the G0 to the G1 phase of the cell cycle, complete replication (S), and go to the G2 phase. Only a chromosome that has completed replication can generate two signals from one centromere, i.e., each chromatid from a chromosome with PCD containing prematurely separated chromatids behaves like a separate chromosome.

No cells in mitosis were observed. The quantitative FISH was performed based on the procedure described in Iourov et al. [20]. The distribution of unscored cells was 16% (not scorable as a result of signal losses due to truncation). Each slide was scored in a blind fashion by an experienced cytopathologist, who was blinded to the diagnosis of each slide (AD vs. control).

The slides were analyzed using an Olympus AX50 epifluorescent microscope (Olympus, Tokyo, Japan) under 1000× magnification. The intensity of the centromere signals in neurons was determined using the CytoVision FISH software (Applied Imaging, Dornach, Germany); pixel mean intensity of the ROI was used, as recommended by the manufacturer. The signals were normalized by the DNA intensity of the whole nucleus. The intensities of the single-spot signals and split-spot signals indicating PCD were measured separately. Based on the fluorescence intensity of the integrated (non-split) centromeric signal, it was concluded whether a duplicated signal represented PCD. If the intensity of fluorescence of a double-spot signal corresponded to twofold the intensity of a single centromeric signal, the two neighboring dots were considered to be a visual manifestation of PCD.

### 2.3. Statistical Analysis

Statistical analysis was performed using the Mann–Whitney test with Statgraph 4.2 software, and differences of *p* < 0.05 were considered significant

## 3. Results

**Figure 1:**
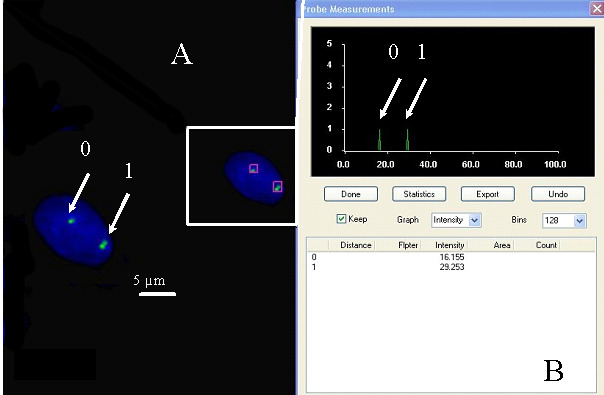
Examples of the quantitative assessment of FISH signals. (**A**) Nucleus with one spot signal of a specific probe for chromosome X (relative intensity—arrow 0) (PCD−) and two spots (PCD+), chromosome X-specific probe (arrow 1). (**B**) The relative fluorescence intensity of the centromeric signals was measured.

**Figure 2:**
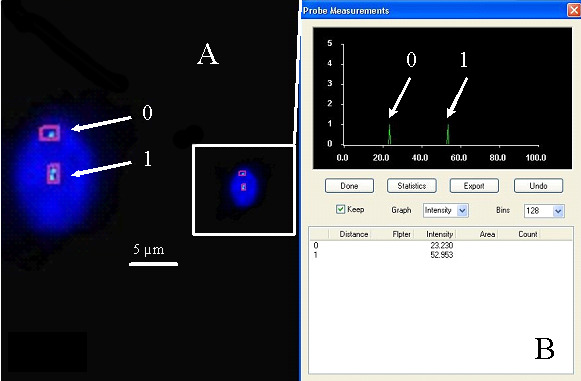
Examples of the quantitative assessment of FISH signals. (**A**) Nucleus with one spot signal of a specific probe for chromosome X (relative intensity—arrow 0) (PCD−) and two spots (PCD+), chromosome X-specific probe (arrow 1). (**B**) The relative fluorescence intensity of the centromeric signals was measured.

**Figure 3:**
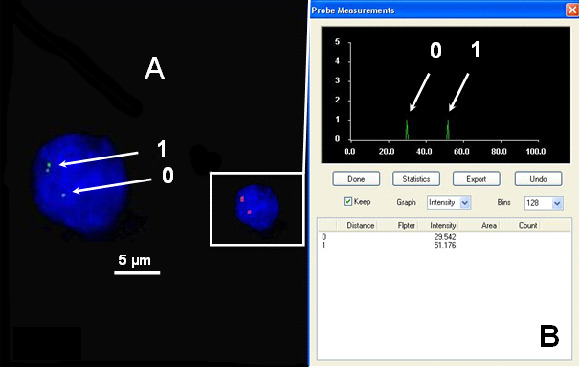
Examples of the quantitative assessment of FISH signals. (**A**) Nucleus with one spot signal of a specific probe for chromosome X (relative intensity—arrow 0) (PCD−) and two spots (PCD+), chromosome X-specific probe (arrow 1). (**B**) The relative fluorescence intensity of the centromeric signals was measured.

**Figure 4:**
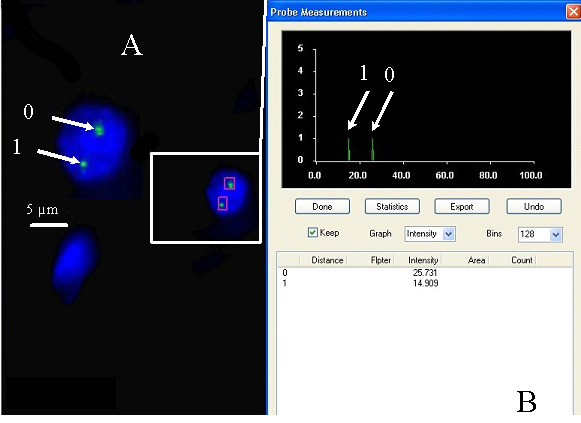
Example of a split signal detected by the quantitative assessment of FISH signals. (**A**) Nucleus with one spot signal of chromosome X-specific probe (relative intensity—arrow 1) (PCD−) and two spots (PCD+), chromosome X-specific probe (arrow 0). (**B**) The relative fluorescence intensity of the centromeric signals was measured.

**Figure 5:**
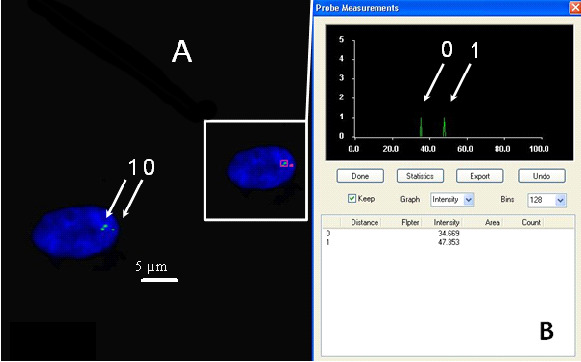
Example of a split signal detected by the quantitative assessment of FISH signals. (**A**) An AD brain neuron cell counterstained with DAPI with signals of chromosome X (PCD−) and two spots like the chromosome X-specific probe (relative intensity of each spot in pixels), characterizing split signals in the centromere. (**B**) DNA histogram obtained by measuring nuclear DNA content, revealing that both signals 0 and 1 have similar fluorescence intensity, indicating that signal 1 represents a split signal rather than PCD.

The relative fluorescence intensities of centromere signals in neuron cells were measured. In females, the diploid neuronal nuclei exhibited two distinct FISH spots for the centromere of each X chromosome, showing that the signals were of similar fluorescence intensity. Nevertheless, in some cells, split signals for one centromere were observed. The fluorescence intensity of each divided FISH spot was similar to the undivided centromere signal. If the fluorescence intensity of the split signals was twice that of the single centromere signal, we concluded that the PCD phenomenon was present in the observed cells (PCD+) (Figure 1, Figure 2, Figure 3 and Figure 4). Conversely, if the joint fluorescence intensity of the divided signals was equal to the fluorescence of a single centromere signal, we represented this as the absence of PCD (PCD−) (Figure 5).

**Figure 6:**
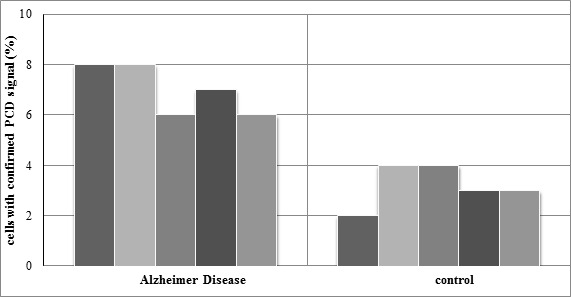
Quantitative assessment of X-chromosome FISH signals in interphase nuclei. AD and control cells with confirmed PCD signals. One hundred interphase nuclei from hippocampal neurons were scored for each sample. The percentage of nuclei displaying PCD,X is significantly higher in the AD cases analyzed as compared with the controls (*p* < 0.01; Mann–Whitney).

After analyzing neuronal nuclei from all AD and control samples, the average frequency of PCD,X in the AD group was found to be 7 ± 1%, whereas in the group of five age-matched female controls, the average frequency of PCD,X was found to be 3.2 ± 0.84% (Figure 6). In both AD and control cases, PCD,X was present on only one of the two X chromosomes in all analyzed nuclei. The results show an almost twofold higher incidence (*p* < 0.01) of PCD,X in the hippocampal neurons of AD patients as compared with the age-matched controls. Additionally, no mitotic cell divisions were observed.

## 4. Discussion

The assessment of FISH spots is a cumbersome process [22,23,24,25,26,27], especially when estimating the PCD phenomenon. Therefore, a novel approach to analyzing FISH spots in chromosome imbalances would be welcomed. Until now, the fluorescence intensity of hybridization (centromeric) spots in chromosomal alterations has not been substantially investigated. Using DNA FISH probes that hybridize to the alpha satellite DNA of the centromeric region provides an advantage for detecting centromeric chromosome (DNA) copy numbers in interphase nuclei [15,22,23,24,27] to evaluate human interphase chromosome instability (CIN) in the brain nuclei as well as in the peripheral tissue cells.

Fukagawa et al. [28] defined the PCD phenomenon in interphase nuclei as a bipartite centromere signal if the distance between two signals was not greater than half of the centromere width, while a single dot-like signal was scored as PCD−. To properly address the X chromosome variation in the CIN in the interphase nuclei, the key question is this: how can we easily and accurately estimate the PCD phenomenon using FISH for the centromeric region and establish a clear distinction regarding chromosomal aneuploidy?

In this study, we present an image system that allows the quantitative analysis of the fluorescence intensity of the centromere region, i.e., the size of individual FISH spots. Because the PCD phenomenon is characterized as a bipartite signal with two separated FISH spots, it is useful to assess whether the quantity of the hybridized alpha satellite probes in each spot corresponds to the expected centromere amount. This approach allows us to make a clear distinction between the PCD phenomenon and split centromere signals. Split centromere signals contain mainly singles with low copy numbers (low intensity), indicating that the bipartite signal stands for a split signal.

Variations of alpha satellite DNA presented in the centromeric regions of all human chromosomes are detectable by the quantitative analysis of FISH for chromosome-specific alphoid DNA probes [29,30]. This approach revealed that one of two centromeric spots in a CAL-51 breast cancer cell was apparently brighter than the other when the homologous chromosomes 7, 11, 17, and 18 were analyzed [31]. This variation in the FISH-signal fluorescence intensity of the spots on the two homologous chromosomes in the nucleus may result from differences in the origin of the homologous chromosomes (paternal and maternal). Furthermore, the quantitative approach indicates that genetic changes in the cell cycle can be assessed by estimating the fluorescent intensity of FISH spots. The intensity of centromeric FISH spots increases during the S phase, displaying a significant increase in the late S phase which is similar to that in the G2 phase, showing that alpha satellite DNA is replicated in the late S phase [31].

Moreover, the interphase nuclei of neurons had one signal per nucleus after hybridization with classical satellite DNA probes for the heterochromatic regions of chromosomes 1, 9, and 16 in 5% to 40% of brain nuclei [20]. The quantitative assessment of FISH signals in interphase cells with one signal displayed approximately two-fold intensity and represented the nuclei with two somatically paired (or over-posed) FISH signals on both homologous chromosomes. These assessments indicated that the mentioned nuclei could not be considered as monosomic.

Additionally, Iourov et al. [20] presented the quantitative assessment of variable X-chromosome FISH signals, allowing for the differentiation of homologous X chromosomes into active and inactive by alphoid DNA heteromorphism. This approach could be helpful in X-chromosome inactivation studies in females with mosaic forms of X chromosome aneuploidy.

In this study, the micro image system was used to estimate the intensity of FISH spots for the X chromosome centromere region (alpha satellite DNA of the centromeric region) by conducting quantitative analysis. This approach can be considered an efficient tool for the accurate and easy determination of CIN in AD interphase nuclei. Although our previous research demonstrated that the X chromosome is vulnerable to the phenomenon of PCD in peripheral blood lymphocytes of AD patients [12], it had remained uncertain whether this effect is also observed in the brain. To the best of our knowledge, this is the first time the analysis of the FISH spot intensity of the centromere region was used to determine the PCD phenomenon in AD hippocampal brain cells. The presence of this pattern of genome instability in both the peripheral blood cells and neuronal tissue supports the potential use of PCD of the X chromosome as a potential biomarker in early diagnosis of AD.

Based on our comprehensive observations, we conclude that a quantitative approach should support the qualitative analysis of FISH centromeric spots in the study of chromosomal alterations in AD. The mere presence of a double signal at the centromere of a single X chromosome is proof that hippocampal neurons have re-entered the cell cycle, DNA has replicated, and premature centromere division has occurred. Furthermore, because no cells undergoing mitosis were seen, it is plausible that the observed AD neurons with PCD might proceed to apoptotic degradation as we previously discussed [14,15,16,17].

An advantage of this approach is that by using the fluoresce centromere labeling method and recording the total fluorescent signal intensity in the centromeric region (being either a single signal or two signals), one can determine whether the cells are in G0 phasis or whether they are starting a new cell cycle. However, it should be taken account that FISH comes with idiosyncrasies that need to be considered carefully in both experimental design and result interpretation. Understanding these limitations, particularly in terms of sample preparation, probe specificity, and resolution, is essential for achieving reliable and meaningful results for diagnostic or prognostic purposes, where consistency and reproducibility are crucial. Furthermore, the small sample size in this paper has several limitations. Using a small dataset may not represent the broader population accurately, because findings from a few specimens may be skewed by individual variability or technical artifacts, making it difficult to extrapolate conclusions to larger populations. Additionally, smaller sample size studies are more susceptible to selection bias due to poorer randomization of the samples. Thus, certain abnormalities may be over- or underrepresented, reducing the ability to characterize rare chromosomal events comprehensively. It would be useful to increase the sample sizes in subsequent studies and provide multicenter samples to extrapolate these obtained conclusions to larger populations with confidence.

## 5. Conclusions

This study presents a novel quantitative technique using fluorescence in situ hybridization to assess X chromosome centromere signals in hippocampal brain tissue from Alzheimer’s disease patients. The results demonstrate a significantly higher frequency of premature centromere division in AD neurons compared with the controls, indicating chromosomal instability. The presence of double FISH signals at the centromere of a single X chromosome suggests cell cycle reentry and DNA replication in AD hippocampal neurons. This technique provides a reliable method for identifying PCD+ signals and contributes to our understanding of chromosomal instability in AD. Taking into account the fact that PCD of the X chromosome has also been demonstrated in peripheral blood lymphocytes, these findings support the potential use of X chromosome PCD as a biomarker for early AD diagnosis and highlight the importance of further research into the mechanisms underlying chromosomal instability in neurodegeneration. Future studies should focus on validating these results in larger cohorts and exploring the therapeutic implications of targeting chromosome instability in AD.

## Data Availability

Data are available upon request.

## Abbreviations

The following abbreviations are used in this manuscript:

AD	Alzheimer’s disease
PCD	premature centromere division
FISH	fluorescence in situ hybridization
PBS	phosphate saline buffer
CIN	interphase chromosome instability
